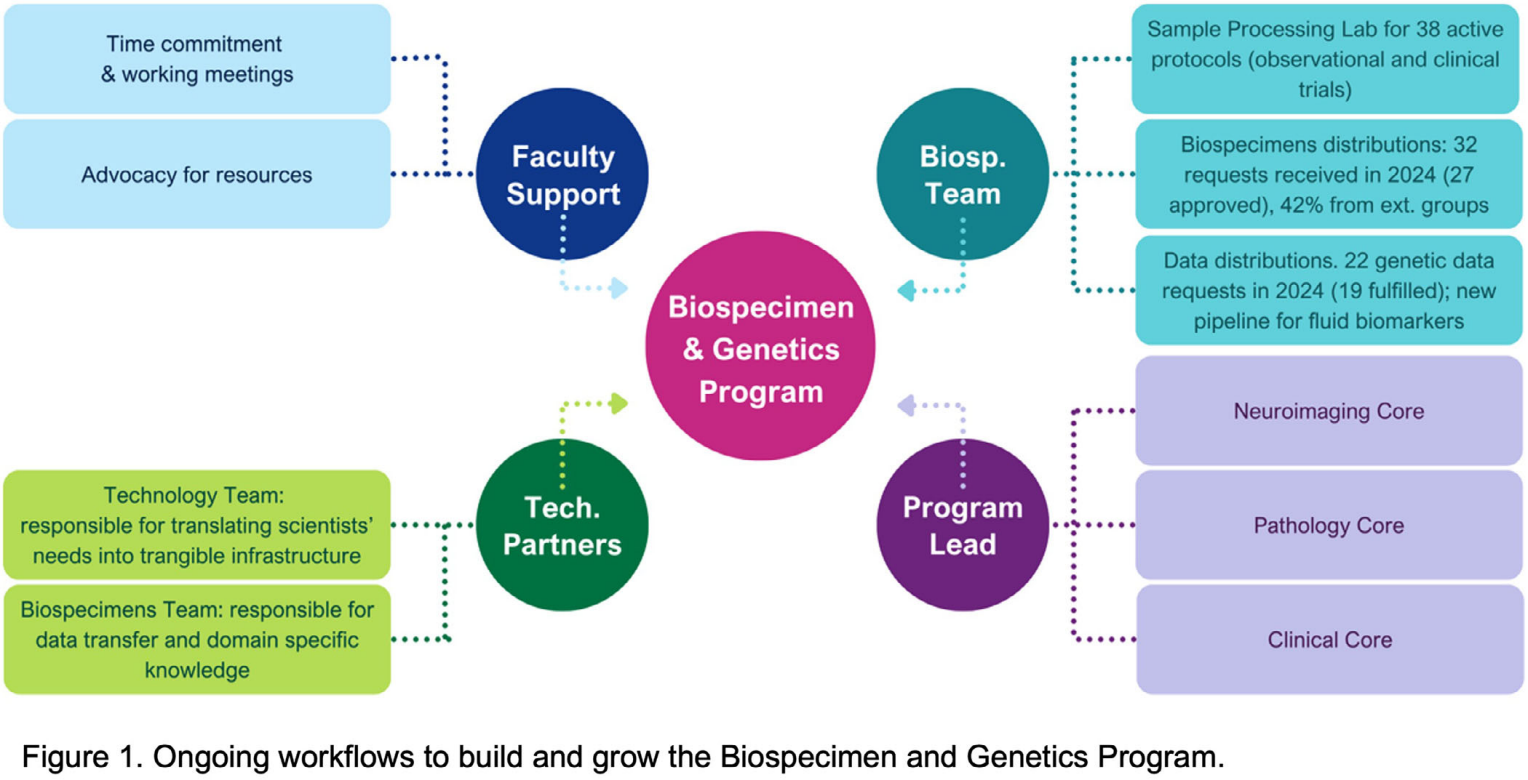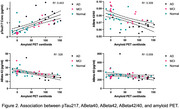# Building infrastructure to support biospecimen and data use as part of the UCSF Memory and Aging Center's ADRC

**DOI:** 10.1002/alz70856_105804

**Published:** 2026-01-08

**Authors:** Argentina Lario Lago, Julia D Webb, Taylor M Young, Rose George, Kristina Noyes, Alexander Shumonov, Claire Yballa, Renaud La Joie, Ana Tyler, Eliana Marisa Ramos, Hilary W. Heuer, Eden V. Barragan, Rowan Saloner, Lawren VandeVrede, Julio C. Rojas, Bruce L. Miller, Gil D. Rabinovici, Kaitlin B Casaletto, Jennifer S. Yokoyama, Adam L. Boxer

**Affiliations:** ^1^ Memory and Aging Center, UCSF Weill Institute for Neurosciences, University of California, San Francisco, San Francisco, CA, USA; ^2^ Memory and Aging Center, Weill Institute for Neurosciences, University of California San Francisco, San Francisco, CA, USA; ^3^ David Geffen School of Medicine, University of California, Los Angeles, Los Angeles, CA, USA; ^4^ Memory and Aging Center, Weill Institute for Neurosciences, University of California, San Francisco, San Francisco, CA, USA; ^5^ Memory and Aging Center, Department of Neurology, Weill Institute for Neurosciences, University of California, San Francisco, San Francisco, CA, USA; ^6^ Department of Neurology, Memory and Aging Center, University of California San Francisco, San Francisco, CA, USA

## Abstract

**Background:**

As one of the largest Alzheimer's Disease Research Centers (ADRC), the Memory and Aging Center (MAC) has strategically developed the infrastructure and personnel of its Biospecimen and Genetics Program (BGP). The BGP supports collection of biospecimens for MAC‐ADRC participants and for 38 other currently‐active research protocols led by 18 investigators by handling collection, storage, and dissemination of human fluid biospecimens and biospecimens‐derived data (biomarker, genetics) at large scale.

Here, we report the efforts that the MAC‐BGP has made to create a program (Figure 1) that facilitates and contributes to AD/ADRD and neurogenerative research nationally and beyond.

**Method:**

During 2024, we continued to be part of the ADRC Fluid Biomarker Initiative (ADRC‐FB) led by the National Centralized Repository for Alzheimer's Disease and Related Dementias (NCRAD). Since we joined the ADRC‐FB in April 2023, we have contributed specimens on over 300 participants. All participants underwent a comprehensive neurobehavioral assessment, and 25% also had amyloid Positron Emission Tomography (PET).

Technologically, we focused our efforts on building BEAM (**B**iospecim**e**n D**a**ta **M**etarepository), a bioinformatics solution for the program. In 2024, we completed work on the foundational database, data pipelines, user interface, and user access control.

**Results:**

The fluid biomarker results generated as part of the ADRC‐FB served a dual purpose for our program:

(1) This dataset has been incorporated into BEAM and has been fundamental to launch its beta version. Currently, the BGP team is using BEAM in day‐to‐day operations which has accelerated data quality checks, management, and dissemination; a small group of investigators are participating in the beta testing program for their work, including data exploration and designing cohorts and experiments that link specimen and biomarker data.

(2) By working closely with the ADRC MAC neuroimaging core, we have reproduced well‐established correlations among plasma pTau217, AB40, AB42, AB42/20, and amyloid PET imaging in a subset of our ADRC cohort (Normal *n* = 18, AD *n* = 15, Mild Cognitive Impairment *n* = 15; Figure 2).

**Conclusion:**

The UCSF‐MAC may serve as an example of how dedicated resources, including engagement from leadership, staff, and technology partners, can promote research by facilitating biospecimen and biospecimen‐derived‐data collection, analysis, and dissemination.